# Ebola Public Health Emergency of International Concern, Democratic Republic of the Congo, 2019

**DOI:** 10.2807/1560-7917.ES.2019.24.29.190718e

**Published:** 2019-07-18

**Authors:** Eurosurveillance Editorial team

**Affiliations:** 1 European Centre for Disease Prevention and Control (ECDC), Stockholm, Sweden

**Keywords:** Ebola, Democratic Republic of Congo, PHEIC

The ongoing Ebola outbreak in the Democratic Republic of the Congo was declared a Public Health Emergency of International Concern by the World Health Organization on 17 July 2017 [1], just short of a year after the start of the outbreak in summer 2018 [2].

The decision followed recent developments such as the occurrence of cases in previously unaffected areas, including the first confirmed case in Goma, a city of almost 2 million people bordering Rwanda, and one case in Uganda. By 16 July 2019, a total of 2,522 confirmed and probably Ebola cases have been reported in this outbreak, 1,698 of them fatal [3]. 

The previous Ebola outbreak in Guinea, Liberia and Sierra Leone from 2014 to 2016 ended with more than 28,000 confirmed, probable and suspected cases and more than 11,000 deaths [4].

More information and the most recent rapid risk assessments by the European Centre for Disease Prevention and Control can be found on a website dedicated to the outbreak [5].
